# Vicarious Menstruation

**Published:** 1861-06

**Authors:** 


*Vicarious Menstruation.*—The N. Y. Medical Press says: "A German girl, aged twenty years, had never menstruated normally; but an ulcer on the inner anterior portion of each tibia would break and bleed freely each month, quite regularly, since she was fourteen years of age. She had spent much of this time under the care of physicians, both in this country and in Germany; had tried changes of air, diet, associations and travelling, both by sea and land, besides medication, but all to no purpose. Dr. McLaury found her, in August, 1859, suffering from periodic pains; for the correction of which, he gave five grains of the iodide of Potassa, with extract of conium; for the menstrual derangement, the ordinary pill of aloes and iron, one night and morning; the bowels moving too freely, after three days but one pill a day was ordered. In just eight days from the time she commenced this treatment, she had the first normal menstruation; and the catamenia continued regular ever since, while the sores on the legs healed completely."

